# Evaluation of the Knowledge, Attitudes, and Practices of Intensive Care Unit Nurses Regarding Eye Care: A Descriptive Study

**DOI:** 10.1111/nhs.70114

**Published:** 2025-04-22

**Authors:** Neslihan Yağmur Gider, Betül Tosun, Ezgi Dirgar

**Affiliations:** ^1^ Department of Nursing, Faculty of Health Sciences Hasan Kalyoncu University Ankara Turkey; ^2^ Faculty of Nursing Hacettepe University Ankara Turkey; ^3^ Department of Midwifery, Faculty of Health Sciences Gaziantep University Gaziantep Turkey

**Keywords:** eye care, intensive care unit, nurse, nursing practice

## Abstract

Eye care is an essential component of nursing practices in intensive care units (ICUs) due to the vulnerability of critically ill patients to ocular complications. The study aims to evaluate ICU nurses' knowledge, attitudes, and practices regarding eye care. This descriptive study, involved 207 ICU nurses from Turkey. Data were collected using the Nurse Introductory Information Form and the Eye Care Clinical Competence Questionnaire. The nurses' mean age was 34.37 ± 7.81. The study found that 54.1% did not record eye problems, and 25% cited excessive paperwork as a barrier. Nurses who followed current studies scored higher on the Eye Care Clinical Competence Questionnaire (*p* = 0.036). Additionally, holding an intensive care certificate significantly improved application sub‐dimension and total scale scores (*p* = 0.020, *p* = 0.023). Educational level significantly impacts ICU nurses' ability to provide eye care, though they face various obstacles in its delivery.

**Summary**

- Nurses with higher education levels and intensive care certificates demonstrated significantly better performance in providing eye care in ICUs.
- Excessive paperwork, insufficient staffing, and low prioritization of eye care were identified as major challenges faced by ICU nurses.
- Specialized training programs, inclusion of eye care in ICU documentation, and mandatory record‐keeping are essential to improving the quality of eye care provided by ICU nurses.

## 1 Introduction

Patients in intensive care units (ICUs) present a more critical population compared to other patient groups due to a range of issues affecting various body systems. These include multiple organ failure, metabolic disorders, impaired consciousness, and the use of muscle relaxants and sedative drugs, all of which can disrupt the eye's protective mechanisms and lead to several complications (Germano et al. 2009; Grixti et al. 2012; Kocaçal and Eşer 2008; Ramírez et al. 2008). One of the most concerning ocular conditions in ICU patients is bacterial keratitis, a severe corneal infection commonly caused by *Staphylococcus aureus* , *Streptococcus pneumoniae* , and *Pseudomonas aeruginosa* . ICU patients, particularly those on mechanical ventilation, are at increased risk due to reduced tear production and compromised ocular defense mechanisms, which facilitate bacterial colonization and infection (Ramani et al. 2021).

In addition to bacterial infections, fungal keratitis is another sight‐threatening complication arising from prolonged ocular surface dryness. Fungal pathogens such as Fusarium, Aspergillus, and Candida can invade the corneal epithelium, leading to severe corneal inflammation and ulceration. These infections are particularly concerning in ICU patients with immunosuppression, prolonged hospitalization, or exposure to contaminated medical equipment (Pulliam et al. 2023; Radhakrishnan et al. 2022). Corneal ulcerations, another major consequence of ocular surface breakdown, can result from bacterial, fungal, viral, or parasitic infections. These ulcers lead to corneal thinning, scarring, and, in severe cases, perforation, which can cause irreversible vision loss (Saritas et al. 2013).

**FIGURE 1:**
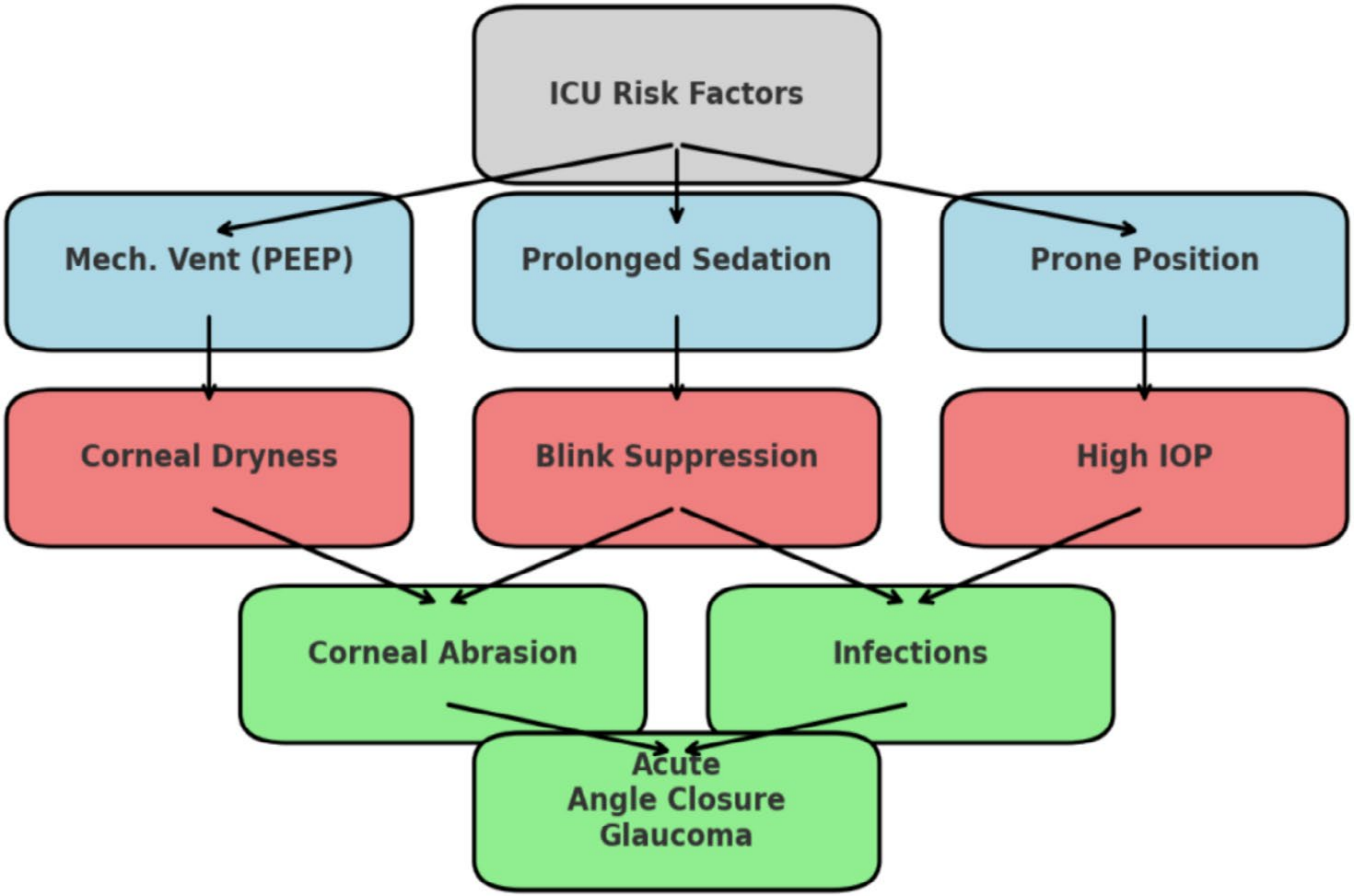
Risk factors for ocular complications in ICU.

ICU patients, particularly those who are unconscious or receiving mechanical ventilation, continuous positive pressure ventilation (CPAP), and sedation, experience impaired ocular protective mechanisms, which exacerbate ocular surface dryness and increase the risk of infections (Awad et al. 2020; Grixti et al. 2012; Hearne et al. 2018; Lahiji et al. 2021; Werli‐Alvarenga et al. 2012). Accompanying chronic diseases, edema, increased capillary permeability, fluid‐electrolyte imbalances, and prolonged prone positioning further contribute to ocular surface disorders, making ICU patients more vulnerable to infections (Ting et al. 2020). Among these, viral keratitis, most commonly linked to herpes simplex virus (HSV) and varicella‐zoster virus (VZV), can lead to corneal scarring and long‐term vision impairment if left untreated (Ting et al. 2020). The combination of impaired consciousness, mechanical ventilation, and a reduced blink reflex in ICU patients further worsens ocular surface dryness, creating an environment conducive to microbial proliferation (Figure 1).

Given these risks, implementing strict eye care protocols, including regular assessments, adequate ocular lubrication, and eyelid closure interventions, is essential in preventing sight‐threatening infections (Lahiji et al. 2021). The preservation of corneal health relies on natural eye protection mechanisms such as tear production, blinking, and complete eyelid closure during sleep (Hearne et al. 2018; Jaafr et al. 2020; Lahiji et al. 2021). The best practices and recommended interventions for eye care in ICU settings are summarized in Table 1.

**TABLE 1:** Eye care best practices in ICU.

Best practice/Intervention	Key findings/Outcomes	References
Standardized eye care protocols	Implementation of standardized eye care protocols significantly reduced the incidence of keratitis from 53.1% to 26.5%, conjunctivitis from 71.9% to 41.2%, dry eye from 68.8% to 29.4%, and corneal ulcers from 71.9% to 35.3%.	Askaryzadeh Mahani et al. 2024
Nurse training programs	Educational interventions led to a significant reduction in ocular surface disorders, with the presence of corneal abnormalities decreasing from 53.3% to 26.7% after implementing an evidence‐based eye care intervention.	Ali et al. 2023
Use of lubricating eye drops and ointments	Application of artificial tears gel reduced the development of dry eye in ICU patients from 21% (with liquid artificial tears) to 9%.	de Araujo et al. 2019
Protective eye coverings	Utilizing polyethylene covers effectively prevented exposure keratopathy, reducing its incidence among ICU patients.	Askaryzadeh Mahani et al. 2024

However, muscle relaxants and sedative medications can impair the blink reflex, leading to rapid tear evaporation, ocular surface dryness, and an increased susceptibility to infections (Awad et al. 2020; Hearne et al. 2018; Lahiji et al. 2021). Mechanical ventilation, specifically high intrathoracic pressure resulting from positive expiratory pressure above 5 cm H_2_O or tight fixation of the endotracheal tube, can further impair the blink reflex and exacerbate ocular surface damage (Grixti et al. 2012; Hearne et al. 2018). Additionally, errors during suctioning procedures in mechanically ventilated patients may further increase the likelihood of ocular surface disorders caused by microorganisms (Płaszewska‐żywko et al. 2021). According to Shaeri et al. 2021), dry eye and corneal abrasion were identified as the most common complications in ICU patients, occurring in 25.8% and 25% of cases, respectively. The mean duration of ocular surface dryness and corneal abrasion was found to be approximately 4 ± 2.93 days following ICU admission (Shaeri et al. 2021). Patients under mechanical ventilation face a particularly high risk of exposure keratopathy, with an incidence of 56% among mechanically ventilated patients (Kousha et al. 2018). Another significant concern in ICU patients is corneal injury, which has been reported to occur, on average, 9 days after admission, affecting more than half of ICU patients in some studies (Werli‐Alvarenga et al. 2011).

## 2 Background

Studies have indicated that nurses working in ICUs often lack awareness of the risks associated with ocular injuries and do not regularly perform ocular screenings (Demirel et al. 2014; Grixti et al. 2012). In fact, a majority of nurses have stated that eye care is considered a low priority (John et al. 2022). This may be due to the primary focus of intensive care nurses on stabilizing the patient's vital functions, with less emphasis placed on eye care as a fundamental nursing intervention within ICUs (Awad et al. 2020; Lahiji et al. 2021). As a result, insufficient attention is given to eye care and the early detection of ophthalmological problems (Demirel et al. 2014; Grixti et al. 2012). Additionally, several studies have revealed that awareness regarding proper eye care among nurses is generally low, leading to application errors resulting from a lack of knowledge (Fashafsheh et al. 2013; Jaafr et al. 2020; Sarıtaş and Fırat 2017). However, research conducted by Demirel et al. (2014) demonstrated a decrease in the incidence of keratopathy following eye care training provided to nurses (Demirel et al. 2014). Similarly, a study by Jaafr et al. (2020) found that a majority of ICU nurses had not received any formal training on eye care, highlighting the need for structured educational programs. On a positive note, Elkasby et al. (2021) reported a significant improvement in nurses' performance regarding eye care for mechanically ventilated patients after the implementation of an eye care package. These findings emphasize the necessity of integrating standardized protocols and training programs into ICU nursing education to ensure optimal eye care practices.

Adding to these challenges, maintaining proper hand hygiene is a fundamental component of infection prevention in ICUs and plays a crucial role in reducing the risk of ocular infections. A recent study emphasized that hand hygiene remains the simplest and most effective measure to control infection risks, particularly during patient care procedures (Arai et al. 2022). Despite its critical importance, compliance rates among healthcare professionals often fall short of recommended standards. Innovative approaches, such as utilizing eye‐tracking technology to analyze and improve hand hygiene adherence, have shown promise in ICU settings (Valek et al. 2023). Adherence to proper hand hygiene protocols, including the use of disinfectant solutions and gloves, is essential during ocular examinations to prevent complications like bacterial keratitis. Emphasizing these practices as part of routine care can significantly reduce the risk of healthcare‐associated infections (HAIs).

Beyond infection control, routine pupil assessment plays a critical role in neurological monitoring in ICU patients. Advanced devices like the NPi‐200 pupillometer provide precise and objective measurements of pupil size and light reflex, enhancing the reliability of assessments (Stutzman et al. 2022). The frequency of pupil assessments should be tailored to each patient's condition, with guidelines recommending regular evaluations at intervals of 4–6 h or as clinically indicated. Comprehensive neurological assessments, including systematic pupil evaluations, are essential for ensuring consistency and accuracy in patient care. According to the American Association of Neuroscience Nurses (AANN), a baseline cranial nerve examination should encompass evaluations of vision, visual fields, and pupil reactions (cranial nerves II and III), among other assessments. This structured approach aids in the early detection of neurological changes, facilitating timely interventions (AANN 2021). Implementing standardized tools and protocols can help healthcare providers identify early signs of neurological deterioration, enabling timely interventions and improving patient outcomes.

Eye care plays a crucial role in optimizing eye health and overall quality of life for patients once they transition out of intensive care settings, as supported by established standards and evidence‐based practices (Płaszewska‐żywko et al. 2021). However, there remains a paucity of information available regarding the knowledge, attitudes, and practices of ICU nurses pertaining to eye care.

The aim of this study is to assess the level of knowledge, attitudes, and practices among ICU nurses regarding eye care, with a particular focus on identifying barriers, training needs, and opportunities for integrating evidence‐based interventions to improve patient outcomes.

## 3 Methods

### 3.1 Design and Setting

Employing a descriptive research design, this study was carried out from October to December 2022. The investigation focused on the ICUs of three publicly run hospitals situated in a specific province located in the southern region of Turkey. In total, the study encompassed 21 individual ICUs within these hospitals.

### 3.2 Sample

The target population for this study comprised all nurses (*N* = 331) actively employed within the ICUs of the designated hospitals within the specified timeframe. To determine the appropriate sample size, the researchers utilized the formula *n* = N.t^2^.p.q/d^2^(N–1) + t^2^.p.q which is commonly employed when the population is known (*p* = 0.50. *q* = 0.50. *d* = 0.05. *t* = 1.96). Based on this calculation, the required sample size was determined to be 141 nurses. Ultimately, the research study was conducted using a sample size of 207 nurses, which accounted for 63% of the total population. Those nurses who chose not to volunteer for participation in the study (*n* = 71) were on leave or duty during the data collection phase (*n* = 23) or had fewer than 6 months of experience in the field of intensive care (*n* = 30) were excluded from the study as they did not fulfill the research criteria.

### 3.3 Data Collection

Data collection for this study involved face‐to‐face interactions with the participants. The researchers utilized the Nurse Introductory Information Form along with the Eye Care Clinical Competence Scale to gather the necessary data. To minimize any potential disruption to the intensive care unit (ICU) workflow, trained nurses coordinated the data collection process. On average, each data collection session lasted approximately 15 min, considering the workload in the ICU unit and ensuring that it did not interfere with the nurses' regular work schedule.

### 3.4 Measurement Instruments

#### 3.4.1 Nurse Introductory Information Form

This form comprises questions aimed at assessing the sociodemographic, professional characteristics, knowledge, and educational status of nurses (Ghaleb et al. 2018; Momeni Mehrjardi et al. 2021; Özkaptan et al. 2021; Vyas et al. 2018).

#### 3.4.2 Eye Care Clinical Competence Questionnaire (ECCCQ)

Eye care clinical competence questionnaire (ECCCQ): In 2021, Özkaptan et al. conducted a Turkish validity and reliability study of the scale initially developed by Ebadi et al. in 2017 (Ebadi et al. 2017; Özkaptan et al. 2021). Although their analyses resulted in a reduction to 26 items, the current study utilized the original 35‐item questionnaire, for which language and content validity had been previously established. The questionnaire consists of three sub‐dimensions: “knowledge,” “attitude,” and “practice.” The first 18 items are multiple‐choice questions designed to assess nurses' knowledge of eye‐related topics. The internal consistency of the knowledge sub‐dimension was assessed using the Kuder–Richardson Formula 20 (KR‐20) reliability coefficient. In this study, the reliability of the knowledge sub‐dimension was found to be at a moderate level according to the KR‐20 coefficient. The attitude sub‐dimension consists of seven items (items 19–25), evaluated on a five‐point Likert scale ranging from “very low” (1 point) to “very high” (5 points), and measures nurses' attitudes towards the risk of eye diseases in unconscious patients undergoing mechanical ventilation. The practice sub‐dimension includes ten items (items 26–35), rated on a frequency scale: “always” (5 points), “usually” (4 points), “sometimes” (3 points), “rarely” (2 points), and “never” (1 point), assessing how nurses perform eye care. The total possible score ranges from 17 to 103 (Ebadi et al. 2017).

Before conducting Confirmatory Factor Analysis (CFA) on the 17 Likert‐type items, data were screened for missing values, outliers, and assumptions of normality. CFA results demonstrated acceptable model fit indices (CMIN/DF = 2.923; GFI = 0.819; IFI = 0.880; TLI = 0.853; CFI = 0.878; RMSEA = 0.097; SRMR = 0.089), and all factor loadings were found to be statistically significant (*p* < 0.001). Cronbach's alpha reliability coefficients were 0.803 for the “attitude” sub‐dimension and 0.892 for the “practice” sub‐dimension, indicating high internal consistency.

### 3.5 Statistical Analysis

The data were analyzed using the IBM SPSS for Windows 26.0 software package, and a significance level of *p* < 0.05 was considered for all statistical decisions. Descriptive statistics, including number and percentage frequencies as well as mean ± standard deviation (SD), were used to summarize the obtained data. The normal distribution of the numerical variables was assessed using the Shapiro–Wilk test. For normally distributed data, the independent samples *t*‐test was utilized to compare two independent groups, while the one‐way analysis of variance (ANOVA) was employed to compare more than two unrelated groups. In cases where variances were found to be homogeneous, the Bonferroni correction post hoc analysis was conducted to determine the source of variance. However, when variances were not homogeneous, the Tamhane's test was utilized. Reliability analyses were performed using the Kuder–Richardson Formula 20 (KR‐20) for the multiple‐choice question items (true/false) and Cronbach's alpha for Likert‐type items. Confirmatory Factor Analysis (CFA) was conducted on Likert‐type items after screening for missing values, outliers, and normality assumptions. Bootstrap Maximum Likelihood method was applied for CFA due to violation of multivariate normality assumptions. A *p*‐value of less than 0.05 was considered statistically significant.

### 3.6 Ethical Considerations

Prior to commencing the study, ethical clearance was obtained from the Non‐Interventional Research Ethics Committee for Health Sciences (Date: 24.10.2022; Decision No: 2022/94). All participants were adequately informed about the study and provided written informed consent, with the assurance that they could withdraw from the study at any point. Throughout the study, adherence to the principles outlined in the Declaration of Helsinki was maintained. Additionally, permission to utilize the scale developed by the original authors was acquired via email.

## 4 Results

The demographic and professional characteristics of the 207 ICU nurses in this study reveal important insights. The average age of participants was 34.37 ± 7.81, with the majority being female (75.8%). Regarding educational background, 75.8% held a bachelor's degree, 14.4% had an associate degree, and 9.8% had postgraduate education. In terms of ICU experience, most nurses had 1–9 years of experience (65.7%), while 29.0% had 10–19 years, and 5.3% had over 20 years of experience. The distribution of work units indicates that most nurses worked in internal medicine ICUs (75.4%), followed by surgical ICUs (20.8%) and neonatal and pediatric ICUs (3.9%). When classified by ICU level, 45.9% were in third‐level ICUs, 44.9% in second‐level ICUs, 5.3% in first‐level ICUs, and 3.9% in neonatal and pediatric ICUs. Workload distribution also varied by shift. During the day, 96.1% of nurses cared for 1–4 patients, while 3.9% managed five or more. At night, 94.2% handled 1–4 patients, and 5.8% managed five or more. A notable finding is that only 47.3% of the nurses had an ICU certification, while 52.7% had not received this training. This underscores the importance of encouraging certification programs to enhance nurses' competencies, particularly in specialized areas such as eye care. These findings provide a structured understanding of ICU nurses' backgrounds and workload, supporting the interpretation of subsequent results (Table 2).

**TABLE 2:** Demographic and professional characteristics of ICU Nurses (*N* = 207).

Descriptive characteristics	Category	Count (*n*)	Percentage (%)
Age (years)	22–34	116	56
M ± SD: 34.37 ± 7.81	35–44	66	31.9
(Min. 22; Max. 53)	Over 45 years old	25	12.1
Gender	Female	157	75.8
	Male	50	24.2
educational status	Associate degree	30	14.4
	Bachelor's degree	157	75.8
	Post graduate	20	9.8
Duration of working in intensive care unit (years)	1–9 years	136	65.7
M ± SD: 7.6 ± 5.7	10–19 years	60	29.0
(Min. 1; Max. 32)	> 20 years	11	5.3
Operating divisions			
	Internal medicine ICUs	156	75.4
	Surgical medicine ICUs	43	20.8
	Neonatal and pediatric ICUs	8	3.9
	3rd step ICUs	95	45.9
	2nd step ICUs	93	44.9
	1st step ICUs	11	5.3
	Neonatal and pediatric ICUs	8	3.9
Number of patients cared for in one shift			
Day shift	Day shift		
M ± SD: 2.87 ± 0.8	1–4	199	96.1
(Min. 1; Max. 8)	5 or more patients	8	3.9
Night shift	Night shift		
Mean: 3.024 ± 0.9	1–4	195	94.2
(Min. 1; Max. 7)	5 or more patients	12	5.8
Working schedule in the ICU	Day shift only	23	11.1
	Night shift only	4	1.9
	Mixed (day and night shifts)	180	87
Do you have intensive care unit training certificate?	Yes I have	98	47.3
	No I have not	109	52.7

Abbreviations: M, mean; Max, maximum; Min, minimum; SD, standard deviation.

The findings on ICU nurses' eye care practices highlight significant trends and challenges. Only 14% of nurses had received formal education on eye care, with training primarily obtained through intensive care certificate programs (6.2%), graduate education (3.9%), or in‐service training (3.9%). This indicates that structured educational opportunities for eye care remain limited, which may contribute to gaps in clinical competency. A crucial finding is that 54.1% of ICU nurses did not record or monitor eye problems, despite the risk of ocular complications in critically ill patients. This suggests that eye care is often overlooked in routine documentation and that integrating standardized eye assessments into ICU workflows may be beneficial.

The most frequently cited barrier to providing adequate eye care was excessive paperwork (25%), followed closely by the perception that eye care is less important than other critical issues (24.2%) and time constraints (21.4%). Additionally, 20.7% of nurses attributed insufficient staffing as a limiting factor, while 8.5% identified a lack of knowledge and skills in eye care as a barrier. These results suggest that systemic challenges, including administrative burden and resource constraints, may hinder effective eye care delivery. Encouragingly, 70% of nurses reported actively following current practices in eye care, which suggests a strong willingness to engage in evidence‐based nursing (Table 3).

**TABLE 3:** Characteristics of ICU nurses' eye care clinical practices (*N* = 207).

Descriptive characteristics	Category	Count (*n*)	Percentage (%)
Do you have a training about eye care?	Yes	29	14
	No	178	86
*n* case of yes, training institution/program	Intensive care certificate program	13	6.2
	Graduate education	8	3.9
	In‐service training	8	3.9
Recording/monitoring status of eye problems	Yes	95	45.9
	No	112	54.1
The most prevalent barrier to providing eye care^a^	Excessive amount of paperwork	35	25.0
	Eye care is less important than other problems	34	24.2
	Lack of time	30	21.4
	Low number of staff	29	20.7
	Not having sufficient knowledge and skills in eye care	12	8.5
Following current practices in eye care	Yes	145	70
	No	62	30

^a^ *n*, folded.

A statistically significant difference was found between nurses' educational status and the knowledge sub‐dimension of the ECCCQ (*p* = 0.035). To determine the source of this difference, a post hoc Bonferroni test was conducted, revealing that nurses with a postgraduate degree had the highest knowledge scores (7.65 ± 2.08), while associate degree holders had the lowest (5.61 ± 3.41). These findings suggest that higher education enhances theoretical knowledge of eye care, which may be associated with more comprehensive academic training and greater exposure to evidence‐based nursing practices (Table 4).

**TABLE 4:** Comparison of descriptive characteristics of intensive care nurses according to the ECCCQ.

Descriptive characteristics	*N*	ECCCQ			
		Knowledge	Attitude	Implementation	Total score
		Mean ± SD	Mean ± SD	Mean ± SD	Mean ± SD
Gender					
Female	157	6.92 ± 2.68	29.41 ± 3.77	39.39 ± 6.75	75.73 ± 10.18
Male; t, p	50	6.96 ± 2.68; 0.069, 0.945	29.18 ± 3.81;0.038, 0.704	39.28 ± 7.48;0.102, 0.919	75.42 ± 10.72;0.190, 0.849
Education status					
Associate degree^a^	30	5.61 ± 3.41	28.71 ± 3.83	38.14 ± 5.91	72.47 ± 10.09
Bachelor's degree^b^	157	7.01 ± 2.59;3.404, 0.035*	29.39 ± 3.81; 0.394, 0.675	39.34 ± 7.02;0.786, 0.457	75.75 ± 10.47;1.631, 0.198
Post Graduate^c^ KW, p	20	7.65 ± 2.08^a‐c^	29.70 ± 3.43	40.85 ± 5.27	78.20 ± 8.40
Having an ICU training certificate					
Yes I have	98	7.08 ± 2.56	29.75 ± 3.56	40.54 ± 6.45	77.37 ± 9.48
No I have not; t, p	109	6.80 ± 2.78; 0.735, 0.463	29.00 ± 3.92;1.441, 0.151	38.31 ± 7.18; 2.338, 0.020*	74.11 ± 10.77; 2.297, 0.023*
Having a training about eye care					
Yes	29	7.41 ± 2.54	29.44 ± 2.97	40.06 ± 5.15	76.93 ± 7.49
No; t, p	177	6.86 ± 2.70; 1.022, 0.308	29.35 ± 3.90; 0.129, 0.897	39.24 ± 7.19; 0.751, 0.456	75.45 ± 10.71;0.917, 0.364
Do you record and monitor the status of eye problems in ICU patients?					
Yes	95	7.20 ± 2.68	29.54 ± 3.64	39.97 ± 6.64	76.72 ± 9.56
No; t, p	112	6.71 ± 2.66; 1.302, 0.194	29.19 ± 3.88; 0.666, 0.506	38.84 ± 7.14; 1.172, 0.243	74.75 ± 10.83;1.373, 0.171
Status of following current practices					
Yes	145	7.15 ± 2.59	29.64 ± 3.50	40.0 ± 6.14	76.79 ± 8.88
No; t, p	62	6.43 ± 2.83; 1.771, 0.78	28.69 ± 4.29; 1.662, 0.098	37.88 ± 8.34; 1.797, 0.076	73.01 ± 12.70; 2.129, 0.036*
Operating divisions					
Internal medicine ICU	156	7.00 ± 2.46	29.35 ± 3.75	39.32 ± 7.09	75.67 ± 10.13
Surgical medicine ICU	43	6.51 ± 3.28	29.25 ± 3.89	39.48 ± 6.65	75.25 ± 11.04
Neonatal and pediatric ICU; KW, p	8	8.00 ± 3.02; 1.218, 0.298	29.87 ± 4.01; 0.090, 0.914	39.62 ± 5.39; 0.016, 0.985	77.50 ± 10.36; 0.160, 0.852
3rd Step ICUs	95	6.78 ± 2.54	28.94 ± 3.89	38.43 ± 7.11	74.16 ± 10.74
2nd Step ICUs	93	7.06 ± 2.66	29.76 ± 3.51	40.49 ± 6.42	77.32 ± 8.96
1st Step ICUs	11	6.36 ± 2.54;0.7510523	29.09 ± 4.67;0.799, 0.496	37.73 ± 9.39;1.624, 0.185	73.18 ± 15.05; 1.791, 0.150
Neonatal and pediatric ICUs; KW, p	8	8.00 ± 3.02	29.35 ± 4.01	39.62 ± 5.39	77.50 ± 10.36

*Note:* Superscript letters (a, b, c) indicate statistically significant differences between groups, determined by post hoc analysis. *p < 0.05.

Abbreviations: KW, Kruskal‐Wallis; t, independent samples *t* test.

ICU nurses who held an intensive care certification scored significantly higher in the implementation sub‐dimension (*p* = 0.020) and total scale score (*p* = 0.023). This finding suggests that structured certification programs enhance practical competency in eye care. However, the lack of a significant difference in knowledge scores (*p* = 0.463) indicates that certification alone may not be sufficient to improve theoretical understanding, highlighting the importance of continuous education. Receiving formal training on eye care did not yield statistically significant differences in knowledge (*p* = 0.308), attitude (*p* = 0.897), implementation (*p* = 0.456), or total scores (*p* = 0.364). This suggests that existing training programs may not be sufficiently comprehensive or may lack practical reinforcement. Nurses who actively followed current eye care practices had significantly higher total scores (*p* = 0.036). However, while those who recorded and monitored eye problems had slightly higher mean scores across all sub‐dimensions, these differences were not statistically significant (*p* > 0.05). This finding suggests that while documentation should be encouraged, it does not directly translate into increased competency unless supported by education and standardized protocols (Table 4).

## 5 Discussion

This study aimed to assess the knowledge, attitudes, and practices of intensive care nurses regarding eye care. A total of 207 nurses participated in the study. The findings revealed that nurses with pre‐licensure education had lower mean scores in the eye care knowledge subscale compared to those with undergraduate and graduate degrees. These results suggest that formal education plays a crucial role in enhancing nurses' theoretical understanding and practical application of eye care in ICU settings. This conclusion aligns with previous studies conducted by Khalil et al. (2019), Jaafr et al. (2020), and Vyas et al. (2018), which emphasize the significant impact of education on nurses' competency in providing eye care. For instance, Jaafr et al. (2020) found that nurses who received structured training demonstrated greater proficiency in preventive eye care strategies, while Vyas et al. (2018) reported that higher educational attainment was directly correlated with improved eye care practices. These findings reinforce the necessity of integrating specialized eye care training into nursing curricula and professional development programs. Although high school‐level graduates are no longer present in the nursing field in our country, there are still nurses operating at this education level. Therefore, it is imperative to implement in‐service and on‐the‐job training programs to address existing deficiencies in eye care practices. To be effective, these training programs should incorporate evidence‐based guidelines, hands‐on workshops, and simulation‐based learning approaches. Furthermore, structured certification programs tailored to ICU nurses should emphasize the importance of proactive eye care interventions.

This study revealed that the majority of ICU nurses did not receive formal education on eye care during their undergraduate or graduate studies. Among those who did receive training, most acquired their knowledge through hospital in‐service training programs, postgraduate education, or intensive care certification courses. Recent research has also shown that ICU nurses' knowledge levels regarding eye care are insufficient, which can negatively impact patient care outcomes (Thomas et al. 2024). Notably, ICU nurses holding an intensive care certificate demonstrated significantly higher mean scores in both the Eye Care Clinical Competence Questionnaire (ECCCQ) application subscale and total scale, indicating a stronger competency in practical eye care applications. Consistent with these findings, Güler et al. (2017) reported that nurses who received eye care training as part of their ICU certification programs implemented protective practices for ocular health more effectively. Recent studies have demonstrated that quality improvement initiatives can play a crucial role in addressing these challenges. For example, a structured intervention program that incorporated standardized eye care protocols, staff education, and routine documentation was shown to significantly improve adherence to best practices and reduce the incidence of ocular complications in ICU patients (Sevgi et al. 2024). Similarly, Momeni Mehrjardi et al. (2021) found that eye care training specifically designed for critically ill patients significantly enhanced nurses' knowledge, attitudes, and practices. Additionally, a study by Pervaiz et al. (2023) demonstrated that ICU nurses who received education on clinical practice standards for eye care in sedated patients showed significant improvements in their knowledge, attitudes, and practices. Furthermore, Khalil et al. (2019) highlighted that deficiencies in eye care among ICU nurses were primarily due to inadequate educational planning within healthcare institutions. These findings underscore the critical need for continuous professional development through in‐service training and certification programs to ensure that ICU nurses are well‐equipped to provide optimal eye care. Given the importance of structured training programs in improving nurses' competency in eye care, it is essential to examine how ICU nursing education is structured in different countries. In Türkiye, ICU nursing education has undergone significant changes in recent years. Previously, nurses with only a high school diploma and vocational training were eligible to work in ICUs. However, a bachelor's degree in nursing (BSN) is now mandatory for ICU nurses. Additionally, the Ministry of Health has made an Intensive Care Nursing Certificate a requirement for nurses working in ICUs. This certification program includes both theoretical and practical training on patient monitoring, mechanical ventilation, infection control, and basic eye care. Although there is no specialized graduate program in eye care for ICU nurses, ocular health is increasingly emphasized in advanced nursing education. Nurses receive training on common ICU‐related eye complications, such as exposure keratopathy and corneal abrasions, as well as preventive strategies like ocular lubrication and eyelid closure. Recent reports indicate that evidence‐based training programs for ICU nurses are being increasingly integrated into education, emphasizing a multidisciplinary approach to preserving eye health (Pervaiz et al. 2023). Collaboration with ophthalmologists is also encouraged when necessary. As awareness of eye health in ICU patients continues to grow, Turkish nursing education is progressively integrating evidence‐based practices to enhance patient outcomes.

In the present study, the researchers found that nurses predominantly adhered to contemporary practices within ICUs, and those who adhered to such practices achieved higher scores in the application sub‐dimension. These findings highlight the importance of ensuring that ICU nurses stay updated with current evidence‐based protocols, as adherence to such practices has been linked to improved patient outcomes, including reduced rates of ocular surface disorders, ventilator‐associated pneumonia, and hospital‐acquired infections. These findings are consistent with prior research investigations (Mlambo et al. 2021; Momeni Mehrjardi et al. 2021; Savcı et al. 2021). Recent studies also emphasize that continuous professional development and structured training programs significantly contribute to the successful implementation of evidence‐based ICU practices (Thomas et al. 2024; Pervaiz et al. 2023). Consequently, endorsing and promoting the adoption of current practices among nurses is imperative. To accomplish this, it is crucial to enhance nurses' awareness by incorporating structured, evidence‐based training programs into undergraduate and postgraduate nursing curricula. This can be achieved by integrating simulation‐based training, case‐based learning, interactive workshops, and interdisciplinary collaborations with ophthalmologists and critical care specialists. Additionally, hospitals should establish standardized protocols for ICU eye care and encourage routine audits to ensure consistent adherence to best practices.

In the present study, several obstacles hindering intensive care unit (ICU) nurses from providing effective eye care to patients were identified. These included excessive paperwork, lower prioritization of eye care compared to other critical tasks, limited time, insufficient personnel, and a lack of knowledge and skills in eye care. These findings align with previous research by John et al. (2022), who reported that inadequate training was a primary obstacle, with 81.94% of participants perceiving eye care as a low priority. Similarly, Vyas et al. (2018) found that nurses commonly cited lack of time, trained personnel, knowledge, and skills as barriers to providing eye care. The substantial volume of paperwork that ICU nurses must manage can detract from the time available for direct patient care, thereby impacting their ability to attend to critically ill patients effectively. Given the specialized nature of ICUs, it is crucial to consider staff and workload planning that accounts for nurses' time allocation for patient care. Additionally, there is a need to address the neglect of eye care among ICU nurses by raising awareness and drawing attention to this important aspect. Recent studies have highlighted the effectiveness of targeted training programs in improving nurses' competencies in eye care. For instance, Momeni Mehrjardi et al. (2021) demonstrated that training nurses based on eye care clinical guidelines for anesthetized patients significantly improved their knowledge, attitudes, and practices. Similarly, a study by Pervaiz et al. (2023) found that implementing clinical practice standards for eye care in sedated patients led to significant improvements in ICU nurses' knowledge and practices.

To overcome these barriers, it is essential to integrate comprehensive eye care training into nursing education and ongoing professional development programs. Emphasizing the importance of eye care in ICU settings and providing nurses with the necessary skills and knowledge can enhance patient outcomes and reduce the incidence of ocular complications.

In the present study, several obstacles hindering intensive care unit (ICU) nurses from providing effective eye care to patients were identified. These included excessive paperwork, lower prioritization of eye care compared to other critical tasks, limited time, insufficient personnel, and a lack of knowledge and skills in eye care. These findings align with previous research by John et al. (2022), who reported that inadequate training was a primary obstacle, with 81.94% of participants perceiving eye care as a low priority. Similarly, Vyas et al. (2018) found that nurses commonly cited lack of time, trained personnel, knowledge, and skills as barriers to providing eye care in the ICU. The substantial volume of paperwork that ICU nurses must manage can detract from the time available for direct patient care, thereby impacting their ability to attend to critically ill patients effectively. Given the specialized nature of ICUs, it is crucial to consider staff and workload planning that accounts for nurses' time allocation for patient care. Additionally, there is a need to address the neglect of eye care among ICU nurses by raising awareness and drawing attention to this important aspect. Recent studies have highlighted the effectiveness of targeted training programs in improving nurses' competencies in eye care. For instance, Momeni Mehrjardi et al. (2021) demonstrated that training nurses based on eye care clinical guidelines for anesthetized patients significantly improved their knowledge, attitudes, and practices. Similarly, a study by Pervaiz et al. (2023) found that implementing clinical practice standards for eye care in sedated patients led to significant improvements in ICU nurses' knowledge and practices. To overcome these barriers, it is essential to integrate comprehensive eye care training into nursing education and ongoing professional development programs. Emphasizing the importance of eye care in ICU settings and providing nurses with the necessary skills and knowledge can enhance patient outcomes and reduce the incidence of ocular complications.

Recent studies suggest that ICU nurses' knowledge, attitudes, and practices regarding eye care remain inconsistent across different ICU settings, regardless of whether they work in internal medicine ICUs, surgical ICUs, or at different levels of intensive care. This study found no statistically significant differences in knowledge, attitudes, or practices among nurses working in these different ICU settings. However, patient profiles and clinical exposure may still influence nurses' eye care awareness and application. The literature highlights differences in eye care awareness depending on ICU type. Alghamdi et al. (2018) reported that nurses working in surgical ICUs exhibited better knowledge of eye care compared to those in internal medicine ICUs, likely due to the higher exposure to post‐operative patients who require prolonged mechanical ventilation and sedation (Ghaleb et al. 2018). Additionally, studies have shown that nurses in internal medicine ICUs may have lower awareness of eye care compared to those in general ICUs, while those working in units where intubated patients require prolonged management tend to have greater awareness of ocular complications (Güler et al. 2017). Despite the lack of statistical differences in this study, ICU levels can influence nurses' exposure to eye‐related complications. First‐level ICUs primarily manage less critically ill patients, focusing on monitoring and supportive treatments rather than prolonged mechanical ventilation or deep sedation. As mechanical ventilation and sedatives are primary risk factors for ocular complications, nurses in these units may have fewer opportunities to develop eye care expertise. Second‐level ICUs handle moderately critical patients, including those requiring non‐invasive ventilation or intermittent mechanical ventilation. Nurses in these units frequently encounter patients with impaired consciousness and reduced blinking reflex, increasing the need for eye care interventions. Third‐level ICUs manage the most critically ill patients, including those under prolonged deep sedation, neuromuscular blockade, and long‐term mechanical ventilation, which dramatically increases the risk of exposure keratopathy and corneal abrasions. Despite these risks, research suggests that eye care is not systematically implemented, even in advanced ICU settings (Lami and Ayed 2023; Afenigus and Asres 2024). The similar levels of knowledge, attitudes, and practices among nurses across different ICU types and levels may be attributed to several factors. Many hospitals implement standardized ICU training, ensuring that all nurses receive similar baseline education, including fundamental eye care concepts. Studies have consistently shown that eye care is often overlooked in general nursing and ICU training programs, limiting the development of expertise in this area (Lami and Ayed 2023). Additionally, eye care practices are not consistently documented in many ICUs, which may contribute to a lack of systematic implementation and reinforcement among nurses (Afenigus and Asres 2024).

## 6 Limitations of the Study

This study focused solely on the perspectives of nurses employed at three specific public hospitals during a designated timeframe. It is important to note that the observations of nurses' actual practices regarding eye care were not included in this study. Furthermore, it is possible that the nurses may have provided accurate, positive, and desirable responses on the data collection forms.

## 7 Conclusion

This study highlights significant gaps in ICU nurses' knowledge, attitudes, and practices regarding eye care. While higher education and specialized certifications were associated with increased theoretical knowledge, they did not necessarily lead to improved clinical application. Additionally, the findings indicate that the absence of standardized protocols, time constraints due to heavy workload, and limited access to structured training remain key barriers to the integration of effective eye care practices in ICU settings. To address these challenges, targeted interventions such as structured education programs, standardized eye care protocols, and enhanced institutional support should be implemented. Ensuring that eye care is recognized as an essential component of ICU nursing practice will contribute to improving both adherence to preventive measures and overall patient outcomes. Strengthening clinical guidelines and fostering a culture that prioritizes evidence‐based eye care can further support ICU nurses in delivering optimal patient care. By integrating these strategies into routine practice, healthcare institutions can overcome existing obstacles, enhance nurses' competency, and improve the quality of eye care in intensive care settings. Future studies should explore the long‐term impact of such interventions and identify additional approaches to further optimize eye care practices among ICU nurses.

## 8 Relevance for Clinical Practice

Continuous professional development, encouragement of specialized certifications, evidence‐based practice tracking, and interdisciplinary collaboration are vital for improving eye care practices in intensive care units. However, challenges such as lack of standardized protocols, gaps in nurse education, and workload constraints may hinder effective implementation.

**FIGURE 2:**
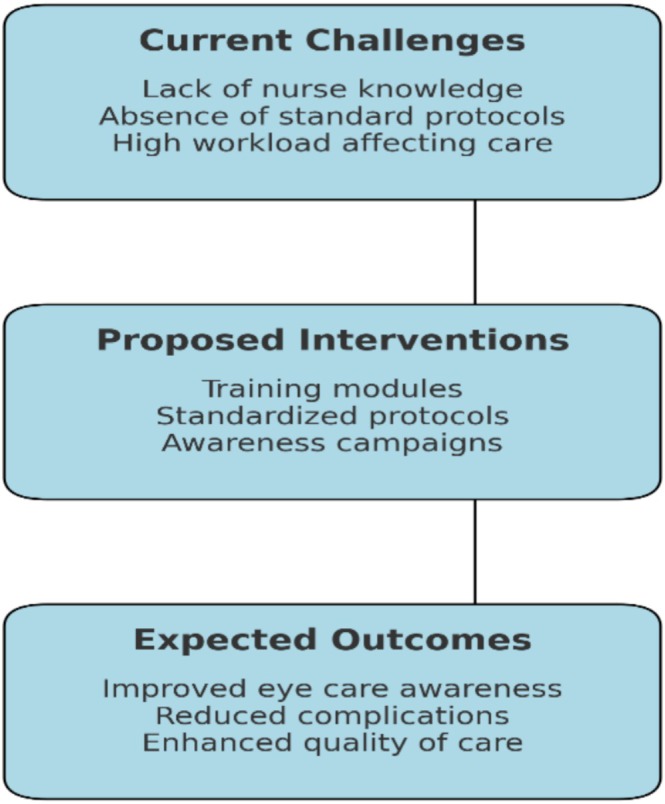
Conceptual framework for translating study results into practical interventions.

As illustrated in Figure 2, addressing these challenges through structured interventions—such as dedicated training modules, standardized care protocols, and awareness campaigns—can lead to improved clinical competency in eye care. By allocating adequate resources and integrating these strategies into routine ICU care, healthcare institutions can overcome existing barriers, empower nurses, and ensure the delivery of optimal eye care practices. Ultimately, these efforts will contribute to reducing ocular complications and enhancing patient outcomes in intensive care settings.

## Author Contributions

**Neslihan Yağmur Gider:** conceptualization, data curation, writing – original draft. **Betül Tosun:** conceptualization, methodology, supervision, writing – original draft. **Ezgi Dirgar:** conceptualization, writing – original draft, methodology, data curation.

## Ethics Statement

The study was started after receiving the required permissions from the Non‐Interventional Research Ethics Committee (Date: 24.10.2022, Decision No: 2022/094), Faculty of Health Sciences, Hasan Kalyoncu University and from the Chief Physician of the Adana Ceyhan State Hospital where the study was implemented. We conducted according to the ethics guidelines set out in the Declaration of Helsinki. All the participant participating in the study were informed about the study, their written/verbal consents were taken, and they were also informed that they could leave the study at any time. The authors declare that they have no competing interests.

## Conflicts of Interest

The authors declare no conflicts of interest.

## Data Availability

The data that support the findings of this study are available from the corresponding author upon reasonable request.
